# Enabling low voltage losses and high photocurrent in fullerene-free organic photovoltaics

**DOI:** 10.1038/s41467-019-08386-9

**Published:** 2019-02-04

**Authors:** Jun Yuan, Tianyi Huang, Pei Cheng, Yingping Zou, Huotian Zhang, Jonathan Lee Yang, Sheng-Yung Chang, Zhenzhen Zhang, Wenchao Huang, Rui Wang, Dong Meng, Feng Gao, Yang Yang

**Affiliations:** 1 Department of Materials Science and Engineering, University of California, Los Angeles, Los Angeles, CA 90095 USA; 2 College of Chemistry and Chemical Engineering, Central South University, 410083 Changsha, China; 3 California NanoSystems Institute, University of California, Los Angeles, Los Angeles, CA 90095 USA; 4 Department of Physics, Chemistry and Biology (IFM), Linköping University, 581 83 Linköping, Sweden; 5 Present Address: College of Chemistry, University of California, Berkeley, CA 94720 USA

## Abstract

Despite significant development recently, improving the power conversion efficiency of organic photovoltaics (OPVs) is still an ongoing challenge to overcome. One of the prerequisites to achieving this goal is to enable efficient charge separation and small voltage losses at the same time. In this work, a facile synthetic strategy is reported, where optoelectronic properties are delicately tuned by the introduction of electron-deficient-core-based fused structure into non-fullerene acceptors. Both devices exhibited a low voltage loss of 0.57 V and high short-circuit current density of 22.0 mA cm^−2^, resulting in high power conversion efficiencies of over 13.4%. These unconventional electron-deficient-core-based non-fullerene acceptors with near-infrared absorption lead to low non-radiative recombination losses in the resulting organic photovoltaics, contributing to a certified high power conversion efficiency of 12.6%.

## Abstract (web summary)

Improving the power conversion efficiency is the main target of the organic solar cell research. Here Yuan et al. develop unconventional electron-deficient-core-based non-fullerene acceptors to achieve both low voltage loss and high current density, leading to a certified high efficiency of 12.6%.

## Introduction

Organic photovoltaics (OPVs) have several advantages such as potential low cost, absorption tunability, mechanical flexibility, and roll-to-roll manufacture capability^1–11^. However, the power conversion efficiencies (PCEs) of OPVs are still inferior to their inorganic counterparts. One of the most significant factors, which hinders their device performance, is the relatively large loss in the open-circuit voltage (*V*_OC_) with respect to the optical gap^12^. While the *V*_OC_ loss can be as low as 0.30 eV in GaAs and slightly higher in c-Si and organic−inorganic hybrid perovskite solar cells (from 0.40 to 0.55 eV), the *V*_OC_ loss for strongly absorbed photons in state-of-the-art OPVs is around 0.6 V or higher^13–15^. In an ideal OPV, maximum *V*_OC_ can be achieved only when sources of voltage losses are limited to an unavoidable radiative recombination of absorption species above the optical gap^16^. However, for the state-of-the-art OPVs, there are strong nonradiative recombination present leading to significant voltage losses, evidenced by electroluminescence quantum efficiency (EQE_EL_) measurements^17–19^. Recombination of radiative charge-transfer (CT) states can also be present due to energetic offsets between the donor (D) and the acceptor (A) components^20–22^. In a very recent work^23^, two design rules are formulated aiming at reducing the voltage loss and increasing the efficiency of OPVs: (1) Small energy offset between the donor and acceptor materials, and (2) high luminescence yield of the lower-gap single component (and hence the blend). Among the materials candidates meeting these design rules, A-D-A-structured non-fullerene acceptors (NFAs) are extremely promising since they exhibit strong intramolecular charge-transfer (ICT) effect, superior energy level tunability, and good molecular crystallinity^24–27^. These features allow NFAs to become potential candidates for achieving an efficient charge separation and low voltage losses simultaneously as per the design rules.

From the aspect of molecular design, synthesis of donor and acceptor materials with complementary absorption profiles intended to maximize the coverage of the solar spectrum has been one of the prerequisites for achieving a high photocurrent. Thus, a variety of narrow optical gap NFAs based on a stronger electron-donating at the central core were carefully designed and synthesized for OPVs^28^. Undoubtedly, it is a successful strategy to elevate the HOMO level reducing the optical gap. Benzotriazole-based conjugated molecules with unique luminescence properties are common building blocks for increasing the photoluminescence quantum yield (PLQY) of the single component^29–32^. The high PLQY indicates efficient radiative recombination pathways, which may result in high electroluminescence yield of the resulting devices. Hence, it attempts to introduce benzotriazole into the central core to form an electron-deficient-core-based fused structure (DAD) for adjusting the optoelectronic properties of the resulting molecules, aiming for a small voltage loss and high performance in devices. By using DAD fused core one could also adopt a relatively planar structure rather than a twisted one and hence, facilitate the electron transfer from the donor to acceptor since the planarized DAD enhances the delocalization of π-electrons. More importantly, a low perturbation of electrons may occur in DAD fused core, which will result in efficient charge transfer^33^. In other words, π-orbital electrons are less likely to be trapped while passing through the fused π-bridge upon introduction of the triazole in this fused system^34^.

In this work, we report a certified high efficiency of single-junction OPVs based on two fused-ring NFA molecules (Y1 and Y2) consisting of dithienothiophen[3,2-b]pyrrolobenzotriazole (BZPT) group with non-halogenated dicyanomethylene derivatives (INIC or INTC). A simple design strategy of covalent nitrogen bridge with adjacent thieno[3,2-b]thiophene and benzotriazole makes it possible to delicately tune the optoelectronic properties of target molecules, resulting in an improvement of optical coverage of near-infrared region (NIR) spectrum. A commercial donor polymer PBDB-T^35^ is selected because of its negligible band offsets with Y1 and Y2. In spite of this small energetic offset, which results in relatively high EQE_EL_ (approximately 0.5 × 10^−4^) and small voltage losses, charge separation is still efficient and offers decent short-circuit current density (over 22 mA cm^−2^), resulting in PCEs of over 13.4% (13.42% for Y1 and 13.40% for Y2). These devices have been certified at the photovoltaic Lab of Newport Corporation, showing a 12.6% efficiency, which is the high efficiency of current single-junction organic cells meeting the ISO 17025 Standards reported so far. This remarkable feature of the presented fused benzotrizole-based system serves as an inspiration to design next-generation NFAs for high-performance OPVs.

## Results

### Characterization of non-fullerene acceptors

To meet the rules mentioned above, our molecule design rational consists of tuning the HOMO levels relatively close to that of the donor and, meanwhile, lowering the LUMO levels to make the absorption spectra of donor material and acceptor materials complementary:

1. Introducing the nitrogen atoms in the center core unit serve as heteroatomic bridges for covalent planarization^36^: this provides stronger electron-donating character and allow for charge carrier mobility in contrast to cyclopentadiene (the center structural unit of the IDTT)^37,38^, thus increasing the HOMO energy levels;
2. Adding a weak electron-withdrawing moiety of 2-ethylhexyl-benzo[d]- [1,2,3]-triazoles segment^39^ in the middle of the central core to form a fused DAD structure: this helps to improve the efficient radiative recombination pathway and enhance the electroluminescence yield of the single components. Based on such a rationale, we are expecting a minimized voltage loss, which enables a high open-circuit voltage and high short-circuit current at the same time.

**Fig. 1:**
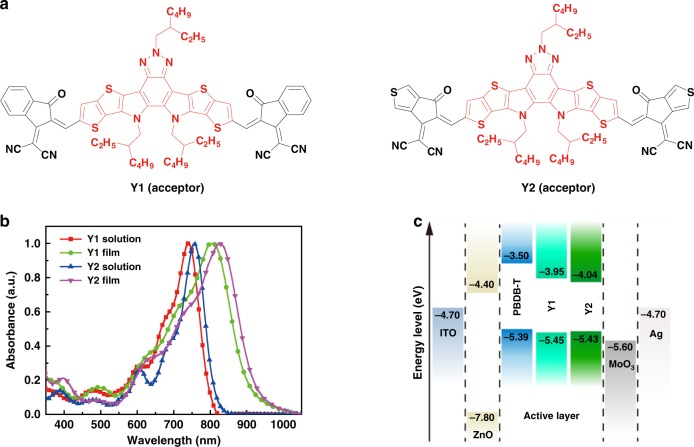
Molecular structure and properties. **a** Chemical structure of the acceptor molecules. **b** Normalized absorption spectra of the acceptors Y1 and Y2. **c** Energy diagrams of the materials used in OPVs. OPV organic photovoltaic

From the materials design point of view, in contrast to previous works^39^, we increase the central fused rings from pentacyclic to heptacyclic, and successfully tune the HOMO energy level to match a small band offset with the PBDB-T, meeting the requirement of the first design rule. In addition, we introduce benzotriazole unit in central core to increase the electroluminescence yield of both the single component, meeting the requirement of the second design rule. From the materials synthesis point of view, the central core that has to be sterically hindered to prevent over aggregation^19^ while maintaining an intramolecular charge transport channel is assumed by the alkyl side chains onto the nitrogen atoms^40^. In contrast to previous designs, it does not need to synthesize spiro-like structures, and this is a good example of non-spiro-like molecule that show excellent performance as an NFA. The chemical structures of Y1 and Y2 are depicted in Fig. 1a, and the detailed synthesis processes are described in Supplementary Figure 1. Nuclear magnetic resonance spectra and high-resolution mass spectrum (MALDI-TOF) of the intermediates, Y1 and Y2 are shown in Supplementary Figs 2−8. Both, Y1 and Y2, can be easily solubilized in common organic solvents, such as chloroform and chlorobenzene at room temperature. Thermogravimetric analysis curves indicate that Y1 and Y2 exhibit excellent thermal stability with decomposition temperatures at 350 °C (Supplementary Fig. 10).

Figure 1b shows the normalized absorption spectra of Y1 and Y2 in solution (chloroform) and in thin films (corresponding optical data in Supplementary Table 1). For the thin films, the absorption peak red-shifts from 738 to 802 nm for Y1 and 758 to 827 nm for Y2. The optical gap of pure Y2 film estimated from the intersection between absorption and emission is about 1.40 eV, which is lower than that of the pure Y1 (*E*_g_ = 1.44 eV, Supplementary Fig. 11). Cyclic voltammetry (CV) measurements (Supplementary Fig. 12) were employed to evaluate the electrochemical properties, showing similar HOMO levels of −5.45 eV for Y1 and −5.43 eV for Y2 due to the identical core center, and a lower LUMO level for Y2 (−4.04 eV compared with −3.95 eV for Y1) due to the stronger electron-withdrawing character of INTC compared with that of INIC (Supplementary Fig. 1). As shown in Fig. 1c, although the ΔHOMO offset between the PBDB-T donor (Supplementary Fig. 9a) and Y1 or Y2 appears to be small (0.07 eV for PBDB-T:Y1 and 0.05 eV for PBDB-T:Y2), efficient charge separation can still be achieved at the bulk heterojunction (BHJ) interface, meanwhile, with suppressed voltage losses. This will be discussed in detail in voltage losses section.

### Device performance of OPVs

**Fig. 2:**
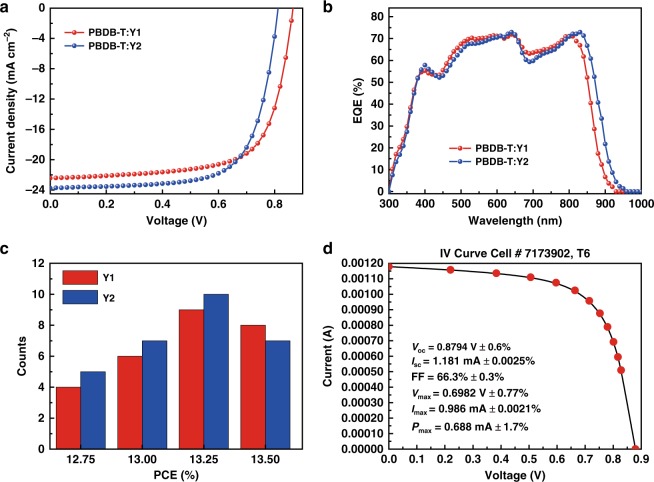
Photovoltaic performance. **a** The *J−V* curves of PBDB-T:Y1 and :Y2 blend solar cells. **b** The EQE curves of PBDB-T:Y1 and :Y2 blend solar cells. **c** Statistical diagram of efficiency of PBDB-T:Y1 and :Y2. **d** Current−voltage parameters of PBDB-T:Y1 device certified (0.0548 cm^2^ device area) by Newport Corp. EQE electroluminescence quantum efficiency

OPVs were fabricated with an inverted device structure of ITO/ZnO/active layer/MoO_3_/Ag (Supplementary Fig. 9b), where PBDB-T was chosen as the donor material. The donor/acceptor ratios in the active layer were carefully optimized (Supplementary Table 2). Figure 2a shows the current density versus voltage (*J − V*) characteristics under the illumination of AM 1.5 G, 100 mW cm^−2^. Table 1 collects the photovoltaic parameters of these OPV devices based on Y1 and Y2, respectively. Meanwhile, the statistical diagram of efficiency of PBDB-T:Y1 and :Y2 is shown in Fig. 2c. Both BZPT-based NFAs, Y1 and Y2, exhibited *J*_SC_ over 22.0 mA cm^−2^ and resulted in PCEs of over 13 % (13.42% for Y1 and 13.40% for Y2, respectively). In Fig. 2b, the external quantum efficiency (EQE) spectra for Y1-based and Y2-based devices demonstrated similar shapes, while the Y2-based device displayed a 25 nm red-shift photocurrent response compared with that of Y1, which was in agreement with the trend of their absorption spectra. For devices with best *I − V* performance, the integrated *J*_SC_ values from EQE spectra with AM 1.5 G reference spectrum of PBDB-T:Y1 and PBDB-T:Y2 were calculated to be 22.14 and 23.18 mA cm^−2^, respectively, consistent with the *J*_SC_ values obtained from the *J−V* curves. Although some previous reports achieved similarly low voltage losses, none of them could keep 60–70% EQE from almost 400 nm to 860 nm^41,42^, which result in the high *J*_sc_ and high PCE of Y1-based and Y2-based devices.

**Table 1:** Photovoltaic performances of PBDB-T:Y1- and PBDB-T:Y2-based OPV devices

Devices^a^	*V*_OC_ (V)	*J*_SC_ (mA cm^−2^)	FF (%)	PCE (%)
PBDB-T:Y1	0.87 (0.87 ± 0.01)	22.44 (21.68 ± 1.07)	69.1 (70.12 ± 0.82)	13.42 (13.22 ± 0.35)
PBDB-T:Y2	0.82 (0.81 ± 0.01)	23.56 (23.12 ± 0.78)	69.4 (70.80 ± 0.70)	13.40 (13.25 ± 0.39)
PBDB-T:Y1*	0.88 ± 0.01	21.55 ± 0.46	66.3 ± 0.80	12.56 ± 0.33

OPV, organic photovoltiac, PCE power conversion efficiency

^a^Certification measurement results are marked with “*”

In order to carefully evaluate the PCE, OPVs based on the PBDB-T:Y1 blend were sent to the PV calibration Lab of Newport Corporation, accredited by A2LA to ISO 17025 Standards, for certification measurements. With the current−voltage parameters shown in Fig. 2d and Table 1, detailed measurements can be found in the section of Methods (Fabrication and measurement of devices). An efficiency of 12.6% was certified with *J*_SC_ of 21.55 mA cm^−2^, a *V*_OC_ of 0.88 V and an fill factor (FF) of 66.3% (Supplementary Fig. 14). The certification confirmed that OPV devices can indeed endure the standard measurement and achieve results relatively close to our regular testing, indicating a reliable and reproducible high performance of our systems.^48^

### Voltage losses and charge separation

In order to evaluate the voltage loss in our system and unravel the nature of its high photovoltaic performance, we investigated voltage losses in both solar cells. The calculated results are presented in Table 2. The total energy loss (Δ*E*) was defined as the difference between the optical gap of blend solar cell and the *V*_OC_. As shown in Table 2, Δ*E* is 0.57 eV for both systems. Specification of the three sources of *V*_OC_ loss follows Equation 1 below:

1 $$\begin{array}{rcl}\Delta E &= E_{\mathrm g} - qV_{{\mathrm{OC}}}\\ &= \left( {E_{\mathrm g} - qV_{{\mathrm{OC}}}^{{\mathrm{SQ}}}} \right) + \left( {qV_{{\mathrm{OC}}}^{{\mathrm{SQ}}} - qV_{{\mathrm{OC}}}^{{\mathrm{rad}}}} \right) + \left( {qV_{{\mathrm{OC}}}^{{\mathrm{rad}}} - qV_{{\mathrm{OC}}}} \right)\\ &= \left( {E_{\mathrm g} - qV_{{\mathrm{OC}}}^{{\mathrm{SQ}}}} \right) + q\Delta V_{{\mathrm{OC}}}^{{\mathrm{rad}},\,{\mathrm{below}}\,{\mathrm{gap}}} + q\Delta V_{{\mathrm{OC}}}^{{\mathrm{non}} - {\mathrm{rad}}}\\ &= \Delta E_1 + \Delta E_2 + \Delta E_3\end{array}$$

**Table 2:** *V*_OC_ loss profile of PBDB-T:Y1- and PBDB-T:Y2-based OPV devices

Devices	*E*_g_ (eV)	$qV_{{{{\mathrm {OC}}}}}^{{{{\mathrm {SQ}}}}}$ (eV)	$qV_{{\mathrm {OC}}}^{{\mathrm{rad}}}$ (eV)	Δ*E* (eV)	Δ*E*_1_ $E_{{\mathrm {gap}}} - qV_{{\mathrm {OC}}}^{{\mathrm {SQ}}}$ (eV)	Δ*E*_2_ $q{{\Delta }}V_{{\mathrm {OC}}}^{{\mathrm {rad}},{\mathrm {below}}\,{\mathrm{gap}}}$ (eV)	Δ*E*_3_ $q{{\Delta }}V_{{{{\mathrm {OC}}}}}^{{{{\mathrm {non}}}}-{{{\mathrm {rad}}}}}$ (eV)
PBDB-T:Y1	1.44	1.17	1.12	0.57	0.27	0.05	0.25
PBDB-T:Y2	1.40	1.13	1.09	0.57	0.27	0.04	0.26

OPV organic photovoltaic

**Fig. 3:**
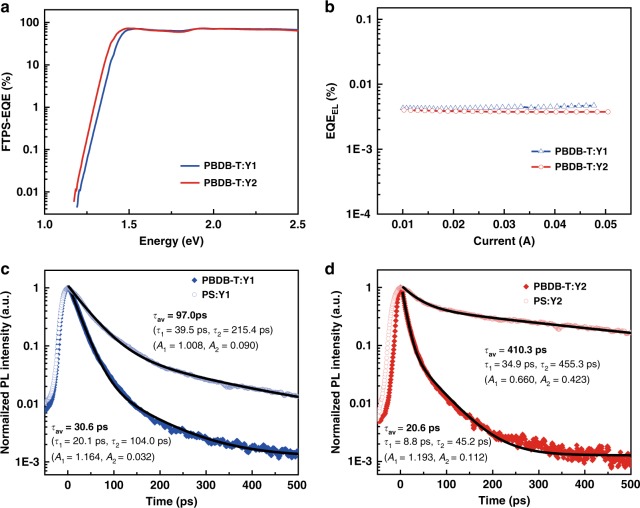
Optical and electrical characterizations. **a** Fourier-transform photocurrent spectroscopy of PBDB-T:Y1 and :Y2 blend solar cell. **b** The electroluminescence quantum efficiency of PBDB-T:Y1 and PBDB-T:Y2 blend solar cells at different injected currents. **c**, **d** Time-resolved for D:A blends and polystyrene blends. Black solid curves are fitting curves for extracting the lifetime ($\tau _{{\mathrm{av}}} = \frac{{{\mathrm{\Sigma }}A_i\tau _i^2}}{{{\mathrm{\Sigma A}}_{\mathrm{i}}\tau _i}}$).

The Δ*E*_1_ is the difference between optical gap and maximum voltage based on the Shockley−Queisser limit (SQ limit), caused by radiative recombination originating from the absorption above the optical gap, typically 0.25 eV or above, unavoidable for any kind of solar cells. Here, $qV_{{\mathrm{OC}}}^{{\mathrm{SQ}}}$ is the maximum voltage based on the SQ limit, where the EQE is assumed to be 1 above the gap and 0 below the optical gap^43^ and the only loss is from the mismatch between radiation received in a narrow solid angle from the sun and omnidirectional radiative recombination. Radiative-recombination loss (Δ*E*_2_) is due to absorption below the optical gap. Here, $V_{{\mathrm{OC}}}^{{\mathrm{rad}}}$ is the open-circuit voltage when there is only radiative recombination, including both the radiative loss mentioned in Δ*E*_1_ and the additional radiative recombination from the absorption below the optical gap due to the nonstep function absorption. For solar cells with steep absorption edges, like inorganic or perovskite solar cells, this term is negligible. However, this part can be very high for OPVs, especially for those with obvious charge-transfer-state absorption. While in our case, the absorption onset of BHJ devices is sharp (Fig. 3a), leading to $q\Delta V_{{\mathrm{OC}}}^{{\mathrm{rad}},{\mathrm{below}}\,{\mathrm{gap}}}$ estimated as low as 0.04 and 0.05 eV, significantly smaller than that observed in typical OPVs and even most small-offset OPVs (Supplementary Table 3). The final part of energy loss stem from the nonradiative recombination (Δ*E*_3_) and could be directly obtained from the equation: $\Delta V_{{\mathrm{OC}}}^{{\mathrm{non}} - {\mathrm{rad}}} = \frac{{kT}}{q}{\mathrm{ln}}\left( {\frac{1}{{{\mathrm{EQE}}_{{\mathrm{EL}}}}}} \right)$. EQE_EL_ results are shown in Fig. 3b. It is notable that both systems show low nonradiative losses of 0.25 and 0.26 eV respectively, which is very low among OPVs reported with such high efficiency (Supplementary Table 3).

We also investigated the PLQY and photoluminescence (PL) quenching for the blend films. Following our previous publication, we blend the insulating polymer polystyrene (PS) with the acceptor materials, to mimic the dispersed morphology in the active layer of the devices^23^. The PLQY values of PS:Y1 and PS:Y2 blends are 5.8% and 3.4% respectively; while those of the PBDB-T:Y1 and PBDB-T:Y2 are 0.1% and 0.3%, respectively, indicating efficient PL quenching. The corresponding PLQY data are summarized in Supplementary Table 4. Although efficient PL quenching indicates efficient charge separation, it also implies additional nonradiative recombination losses, which are to be further decreased. In an ideal case, the PL quenching is expected to happen at the short-circuit conditions, rather than the open-circuit conditions. Efficient PL quenching is further confirmed by the PL decay measurements (Fig. 3c, d), which show shortening of the PL lifetime in the active layers for both Y1 and Y2 blends. Consistent with our and other’s previous reports^23,44^, the timescale on which the PL quenching occurs is very slow compared with traditional fullerene-based devices (those with large energetic offset between the donor and acceptor materials).

### Film morphologies

The molecular packing characteristics of Y1 and Y2 in thin film blend with polymer PBDB-T were investigated using grazing incidence wide-angle X-ray scattering (GIWAXS)^45^. 2D GIWAXS patterns and their corresponding intensity line-profiles in out-of-plane (OOP) and in-plane (IP) directions are presented in Supplementary Fig. 15a and Fig. 16, respectively. PBDB-T:Y1 and PBDB-T:Y2 blends show similar molecular packing. In both blends, a broad peak corresponding to the π−π stacking was observed along the out-of-plane direction at *q* = 1.7 Å^−1^, which benefit efficient photon absorption and charge transport.

The morphologies were further investigated by atomic force microscopy (AFM) and transmission electron microscopy (TEM). As shown in Supplementary Fig. 15b, the formation of obvious ordered nano-fibrillar structures of active layer films were observed using AFM, accompanied with a smooth root-mean-square roughness (*R*_q_) of 1.22 and 1.60 nm on a scan area of 1 μm × 1 μm for Y1- and Y2-based blends, respectively. The phase separation was further visualized using TEM with suitable domain sizes in both blends (Supplementary Fig. 15c), indicating an ideal phase separation for the optimized balance between charge transportation pathway and interface recombination. As a consequence of the orientation, both polymer and non-fullerene molecules in the active layer are observed to closely stack. The lateral charge transportation was also proven to be efficient with space charge limited current (SCLC) method^46^, with the average hole and electron mobilities calculated and shown in Supplementary Fig. 18. Hole mobilities of the PBDB-T:Y1 and PBDB-T:Y2 blend films were measured to be 1.56 × 10^−3^ cm^2^ V^−1^ s^−1^ and 2.59 × 10^−3^ cm^2^ V^−1^ s^−1^, respectively, and electron mobilities were measured to be 3.04 × 10^−4^ cm^2^ V^−1^ s^−1^ and 2.08 × 10^−4^ cm^2^ V^−1^ s^−1^, respectively. These results reflect that the morphology of this system is efficient for charge transport.

## Discussion

In summary, we demonstrate that the two NIR-absorbing multifused benzotrizole-based NFAs, Y1 and Y2, blending with a commercialized donor PBDB-T can achieve a high PCE of 13.42% and 13.40%, respectively. The high performance was enabled by the relatively high EQE_EL_ of approximately 0.5 × 10^−4^, which indicates low nonradiative recombination loss of the blend. In addition, despite small donor−acceptor energy offsets, highly efficient charge generation efficiencies have been shown for both PBDB-T:Y1 and PBDB-T:Y2 blend films. Moreover, a homogeneous and nanophase-segregated structure with an optimized size and preferred orientation are also observed in blend films, which contributed to the efficient charge separation and transport. These results are achieved using a simple design strategy by incorporation of nitrogen acting as a bridging atom between adjacent electron-donor (thieno[3,2-*b*]thiophene) segment and weak electron-deficient segment (benzotriazole) (DAD fused core), leading to strong absorption in Vis−NIR region. A certified high efficiency of single-junction OPVs meeting the ISO 17025 Standards was achieved at 12.6% based on PBDB-T:Y1 devices. This work provides a rational route to a delicate design with relatively tight spacing using the benzotrizole-based DAD structure as central core towards high-performance NFAs.

## Methods

### UV−Vis absorption spectra and cyclic voltammetry measurement

UV−Vis absorption spectra were recorded on the SHIMADZU UV-4100 spectrophotometer. For the solid-state measurements, Y1 and Y2 solutions in chloroform were spin-coated on quartz plates. The CV results were obtained with a computer-controlled CHI 660E electrochemical workstation using polymer or NFA films on platinum electrode (1.0 cm^2^) as the working electrode, a platinum wire as the counter electrode and Ag/AgCl (0.1 M) as the reference electrode in an anhydrous and argon-saturated solution of 0.1 M of tetrabutylammonium hexafluorophosphate (Bu_4_NPF_6_) in acetonitrile at a scanning rate of 50 mV s^−1^. Electrochemical onsets were determined at the position where the current started to rise from the baseline.

### Photoluminescence and electroluminescence spectra

PL and EL spectra were measured using a light guide positioned close to the sample. The excitation light of PL measurement was peaked at 530 nm and a 650 nm-long-pass filter was used during the measurement. The bias of EL measurement was applied on the devices using a Keithley 2400 SourceMeter. The detector was a Newton EM-CCD Si array detector at −60 °C with a Shamrock SR-303i spectrograph from Andor Tech. PLQY values were obtained using an integrating sphere.

### Atomic force microscopy(AFM) and transmission electron microscopy(TEM)

The morphologies of the polymer/acceptor blend films were investigated by AFM (Bruker Dimension Fast Scan Scanning Probe Microscope) in contact mode using a 1 and 5 μm (Supplementary Fig. 17) scanners. Samples for the TEM measurements were prepared as following: The active layer films were spin-casted on ITO/poly(3,4-ethylenedioxythiophene) poly(styrenesulfonate) (PEDOT:PSS) substrates, and the substrates with active layers were submerged in deionized water to make the active layers floats onto the air−water interface. Then, the floated films were picked up on an unsupported 200 mesh copper grids for the TEM measurements. TEM measurements were conducted with a T12 cryo-electron microscope.

### Grazing incidence wide-angle X-ray scattering measurements(GIWAXS)

GIWAXS measurement was performed at Advanced Light Source, Lawrence Berkeley National Laboratory on the 7.3.3 beamline. All the samples were deposited on the silicon wafer with 100 nm silicon oxide. Samples were irradiated by 10 keV at a fixed X-ray incident angle of 0.12° with an exposure time of 3 s. All measurements were conducted under a helium atmosphere to reduce air scattering.

### Fabrication and measurement of devices

The ITO glass was precleaned in an ultrasonic bath of detergent, deionized water, acetone and isopropanol, and UV-treated in ultraviolet–ozone chamber (Jelight Company, USA) for 15 min. A thin layer (30 nm) of ZnO was spin-coated onto the ITO glass by sol−gel method and baked at 200 °C for 60 min. The polymer PBDB-T:Y1 or Y2 (D:A = 1:1, 16 mg mL^−1^ in total) were dissolved in chlorobenzene (CB) and 1-chloronaphthalene (CN) (0.8 %, v/v) overnight (80 °C) and spin-cast at 3000 rpm for 60 s onto the ZnO layer. The thickness of the photoactive layer is about 100 nm measured by Ambios Technology XP-2 profilometer. A bilayer cathode consisting of MoO_3_ (15 nm) capped with Ag (150 nm) was thermal evaporated under a shadow mask with a base pressure of ca. 10^−5^ Pa. Finally, top electrodes were deposited in a vacuum onto the active layer. The active area of the device was 0.1 cm^2^. The certified cells have the area of 0.0548 cm^2^, which is defined by a metal mask with an aperture aligned with the device area.

*J* – *V* characteristics of photovoltaic cells were taken using a Keithley 2400 source measure unit under a simulated AM 1.5 G spectrum with an Oriel 9600 solar simulator. We also conducted both forward and backward scans of PBDB-T:Y1, which yielded identical results (Supplementary Fig. 13). EQEs were measured using an integrated system (Enlitech, Taiwan) and lock-in amplifiers with a current preamplifier under short-circuit conditions.

Asymptotic *P*_max_ protocol, which was proposed by the National Renewable Energy Laboratory’s (NREL) Cell and Module Performance group and accredited to ISO 17025 Standards, can be taken as a reliable evaluation method for the thin film photovoltaics. The optimized device (PBDB-T:Y1) with the PCE of 13.13% (*V*_OC_ of 0.87 V, *J*_SC_ of 21.90 mA cm^−2^ and FF of 69.72%) was sent to an accredited solar cell calibration laboratory (Newport Corp.) for certification. Current−voltage parameters were measured under a 13-point IV sweep configuration wherein the bias voltage (current for *V*_OC_ determination) is held constant until the measured current (voltage for *V*_OC_) is determined to be 0.05% level.

### EQE_EL_ measurement

EQE_EL_ values were obtained from an in-house-built system including a Hamamatsu silicon photodiode 1010B, a Keithley 2400 SourceMeter to provide voltage and record injected current, and a Keithley 485 Picoammeter to measure the emitted light intensity.

### FTPS-EQE measurement

FTPS-EQE was measured using Vertex 70 from Bruker Optics, equipped with a quartz tungsten halogen lamp, quartz beam splitter and external detector option. A low-noise current amplifier (SR570) was used to amplify the photocurrent produced on illumination of the photovoltaic devices with light modulated by the Fourier-transform infrared spectroscope (FTIR). The output voltage of the current amplifier was fed back into the external detector port of the FTIR, to be able to use the FTIR’s software to collect the photocurrent spectrum.

### Optical gap calculation

We determined the optical gaps from the intersection between normalized emission and absorption spectra^47^.

### Hole mobility and electron mobility measurements

The hole-only or electron-only diodes were fabricated using the following architectures: ITO/poly(3,4-ethylenedioxythiophene):poly(styrenesulfonate) (PEDOT:PSS)/active layer/ gold (Au) for holes and ITO/ZnO/active layer/Al for electrons. Mobilities were extracted by fitting the current density–voltage curves using the Mott–Gurney relationship (SCLC). The mobilities were obtained by taking current−voltage curves and fitting the results to a space charge limited form.

### Time-resolved photoluminescence measurement

The time-resolved PL measurements were performed at room temperature. A wavelength-tunable mode-locked Ti:Sapphire laser was used as the excitation source, which has a repetition rate of 76 MHz and a pulse duration of 2 ps. Here, the laser wavelength was set at 725 nm. PL emission induced by the pulsed excitation laser was detected by a Hamamatsu streak camera combined with a single-grating monochromator.

## Supplementary information

Supplementary Information

## Data Availability

The data that support the findings of this study are available from the corresponding author upon reasonable request.
