# Author Correction: Enabling low voltage losses and high photocurrent in fullerene-free organic photovoltaics

**DOI:** 10.1038/s41467-019-09428-y

**Published:** 2019-04-03

**Authors:** Jun Yuan, Tianyi Huang, Pei Cheng, Yingping Zou, Huotian Zhang, Jonathan Lee Yang, Sheng-Yung Chang, Zhenzhen Zhang, Wenchao Huang, Rui Wang, Dong Meng, Feng Gao, Yang Yang

**Affiliations:** 1 Department of Materials Science and Engineering, University of California, Los Angeles, 90095 Los Angeles, CA USA; 2 College of Chemistry and Chemical Engineering, Central South University, 410083 Changsha, China; 3 California NanoSystems Institute, University of California, Los Angeles, Los Angeles, CA 90095 USA; 4 Department of Physics, Chemistry and Biology (IFM), Linköping University, 581 83 Linköping, Sweden; 5 College of Chemistry, University of California, Berkeley, CA 94720 USA

Correction to: *Nature Communications* 10.1038/s41467-019-08386-9, published online 04 February 2019

The original PDF version of this Article contained an error in the Additional information section, which incorrectly included the statement ‘This is a U.S. Government work and not under copyright protection in the US; foreign copyright protection may apply 2019’. This has been removed from the PDF version of the Article. The HTML version was correct from the time of publication.

