# Supplementary material for: Enabling low voltage losses and high photocurrent in fullerene-free organic photovoltaics
Source: Nat Commun. 2019 Feb 4;10:570. doi: 10.1038/s41467-019-08386-9 (PMC6362024; doi:10.1038/s41467-019-08386-9)
Supplement: Supplementary file 1 — Supplementary Information [file 41467_2019_8386_MOESM1_ESM.pdf]

Supporting Information

Enabling low voltage losses and high photocurrent in fullerene-free organic photovoltaics

Yuan, et al.

Supplementary Figures

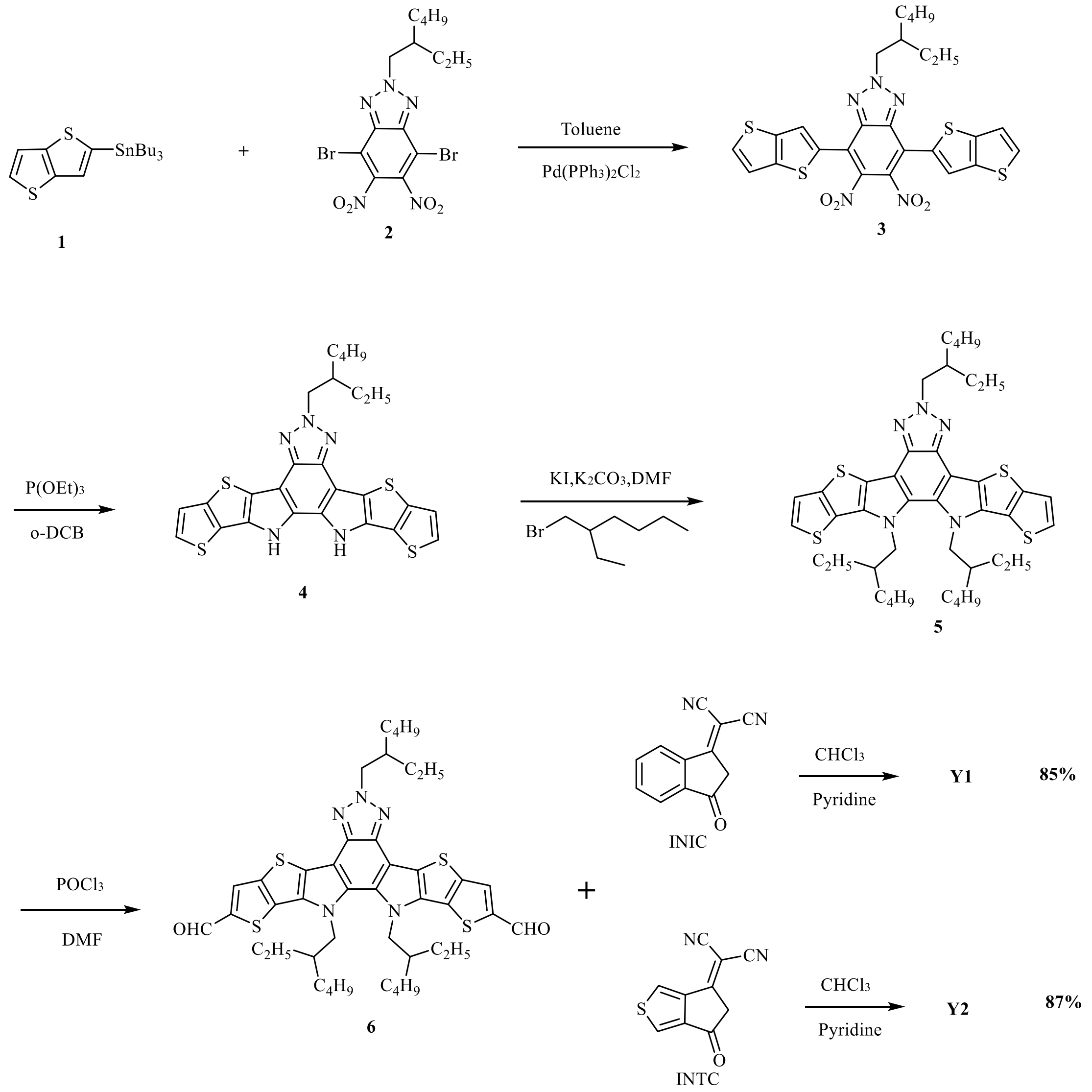

Supplementary Figure 1: Synthetic routes of Y1 and Y2.

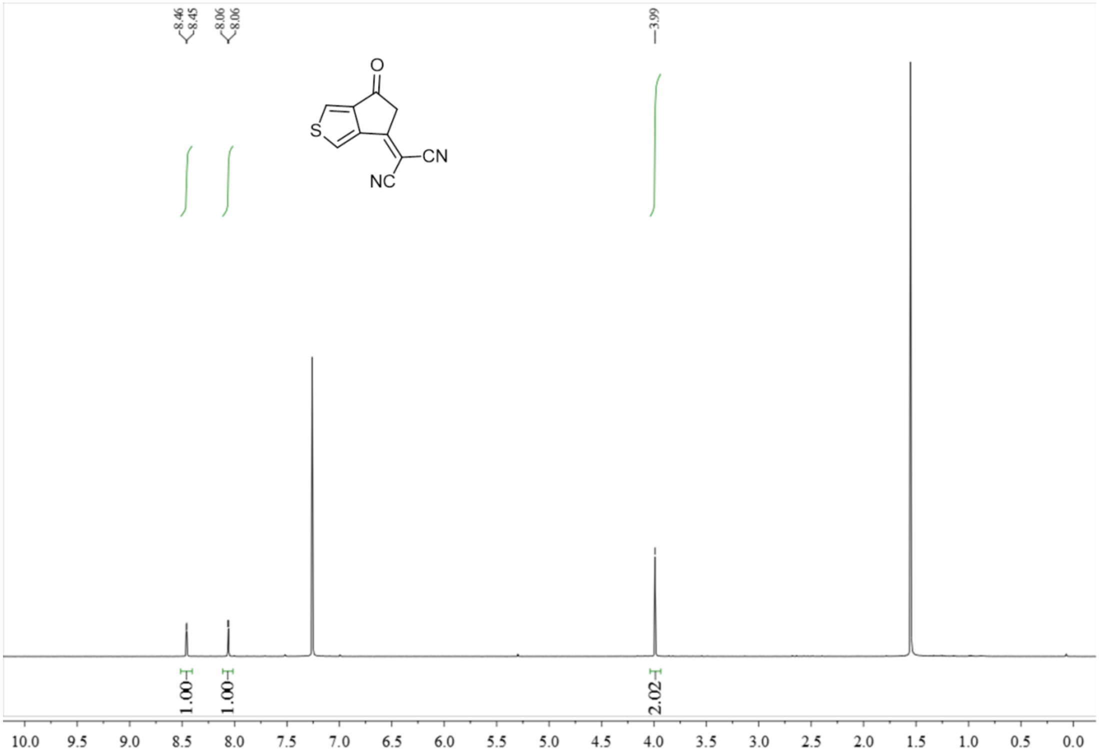

Supplementary Figure 2: ^{1}H NMR spectrum of 2-(6-oxo-5,6-dihydro-4*H*-cyclopenta[*c*]thiophen-4-ylidene)-malononitrile (INTC).

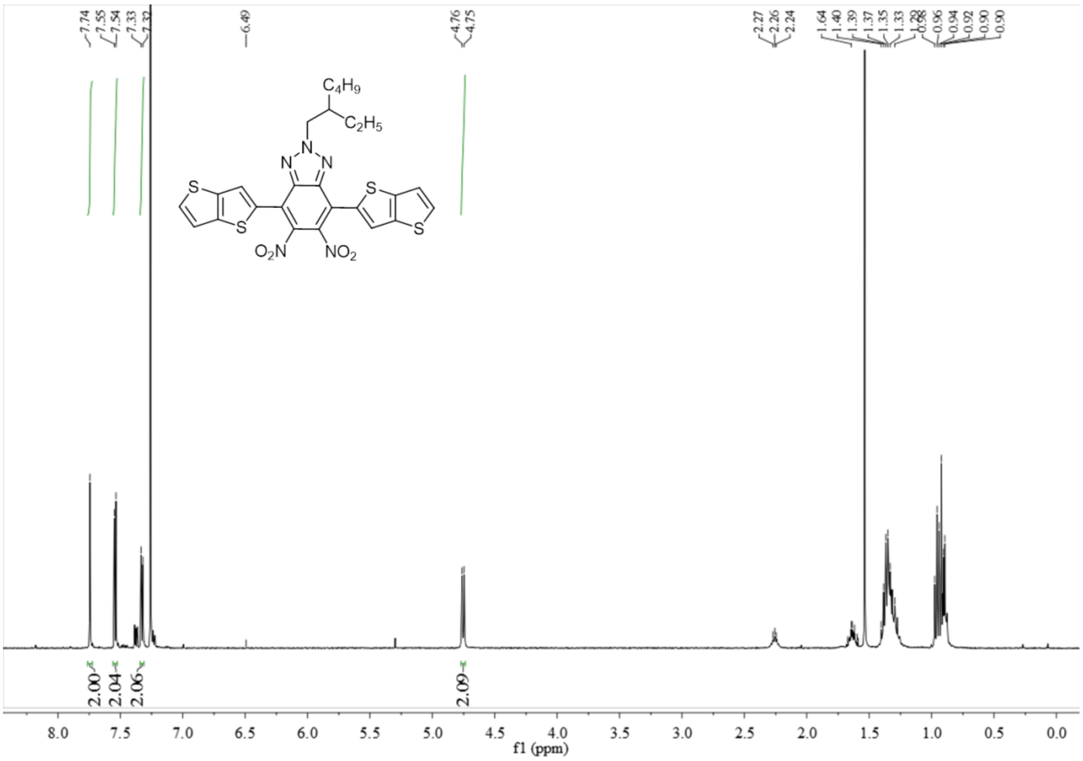

Supplementary Figure 3: ^{1}H NMR spectrum of compound 3.

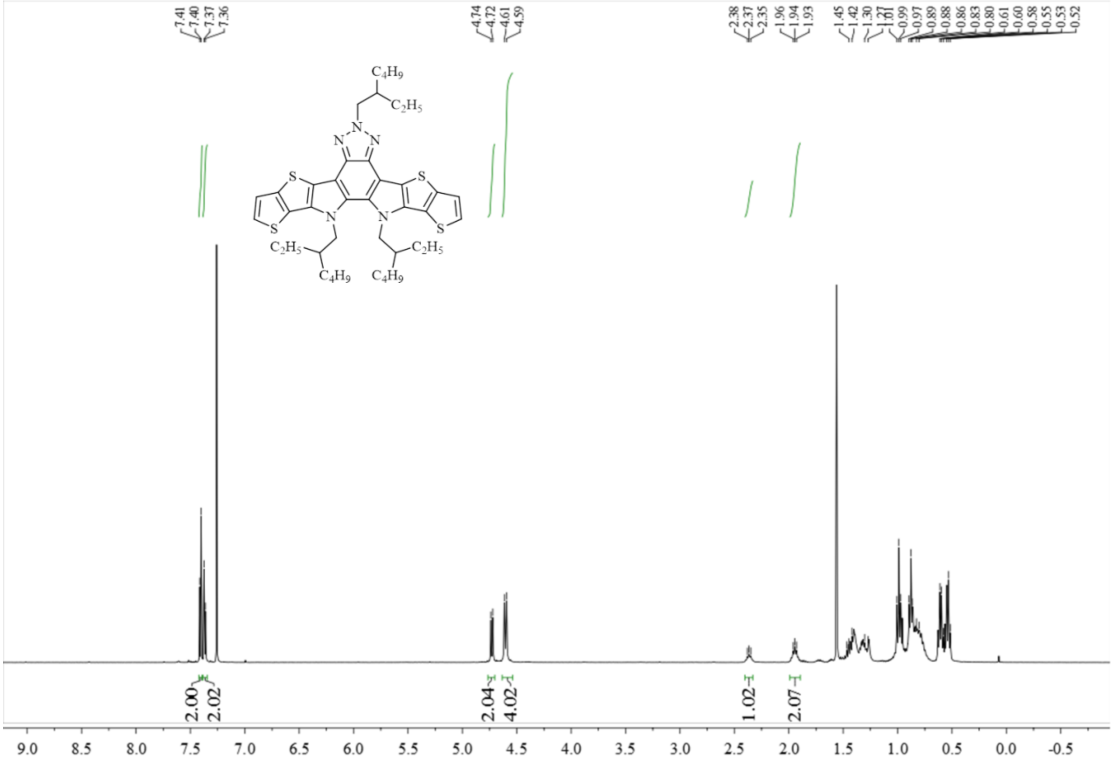

Supplementary Figure 4: ^{1}H NMR spectrum of compound 5.

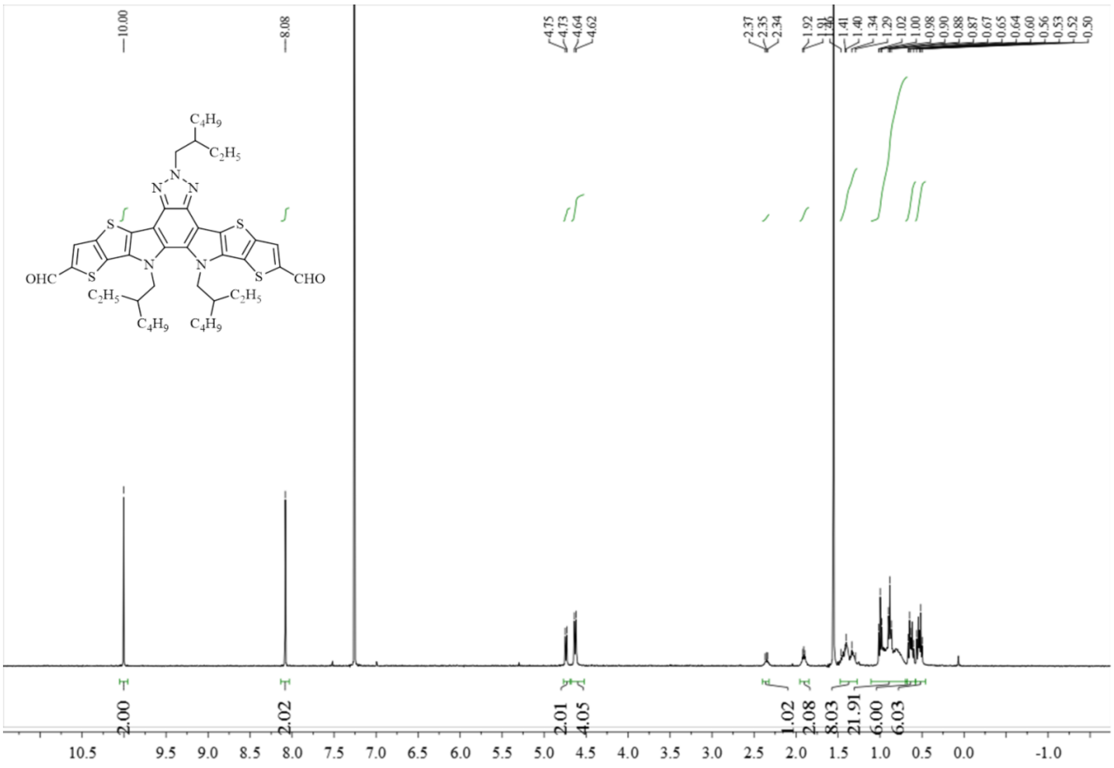

Supplementary Figure 5: ^{1}H NMR spectrum of compound 6.

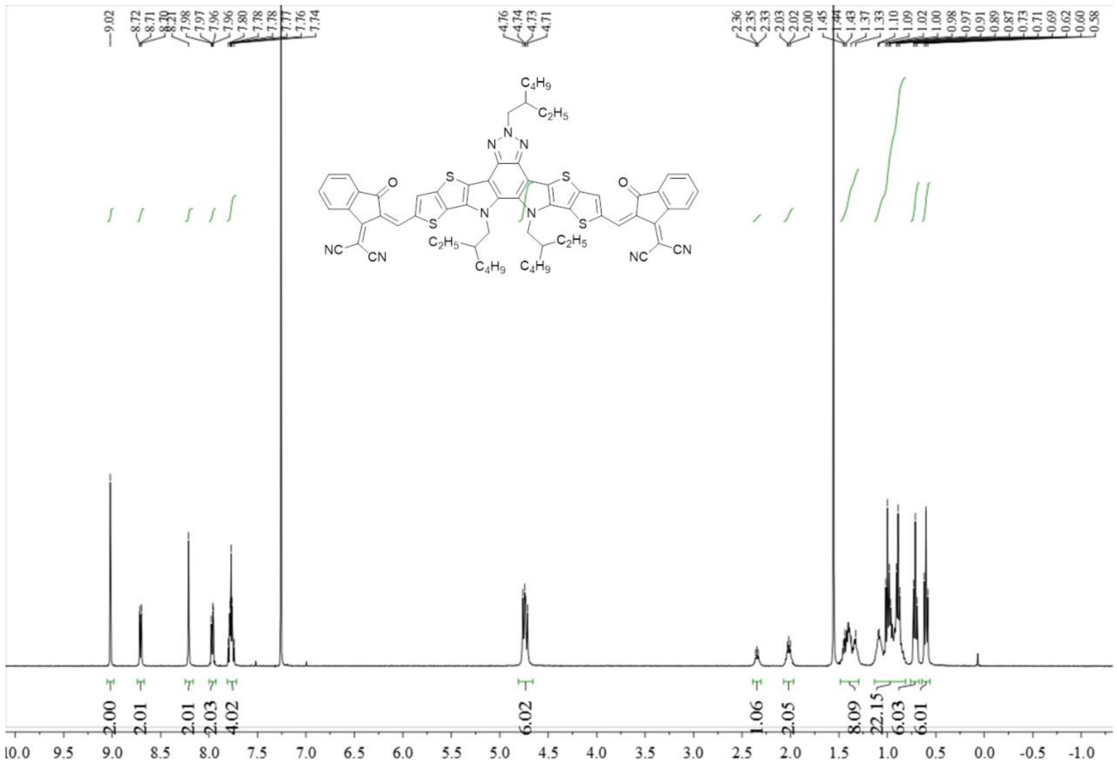

Supplementary Figure 6: ^{1}H NMR spectrum of Y1.

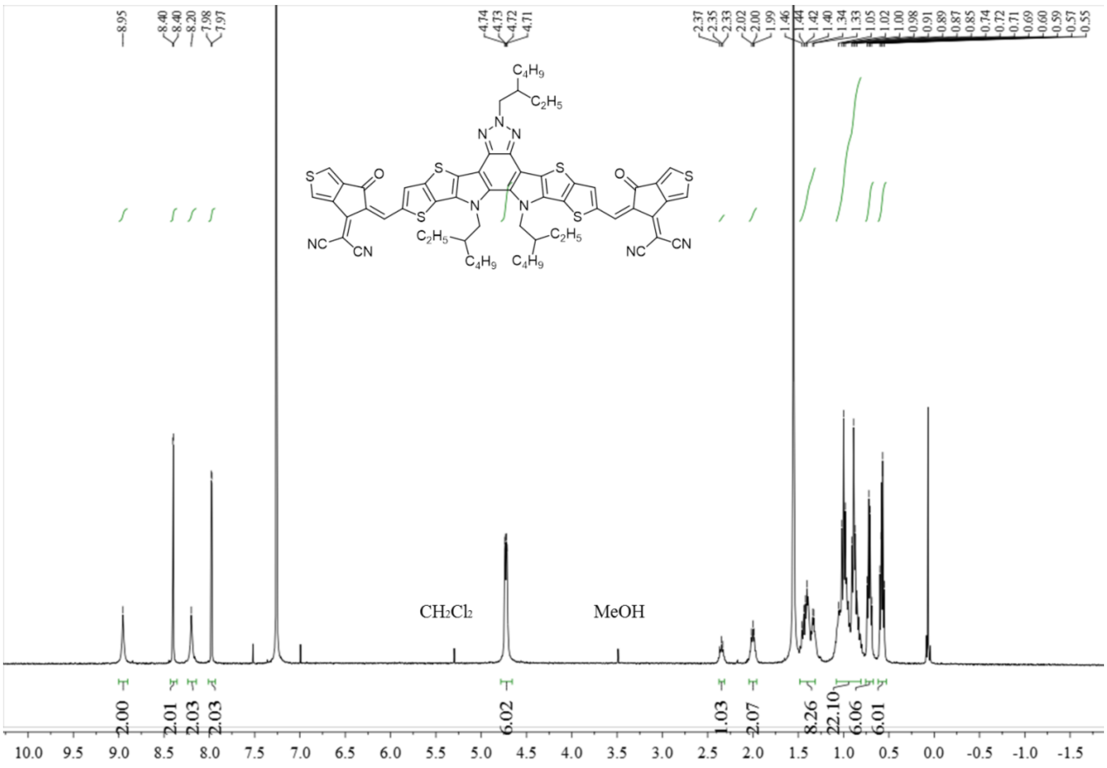

Supplementary Figure 7: ^{1}H NMR spectrum of Y2.

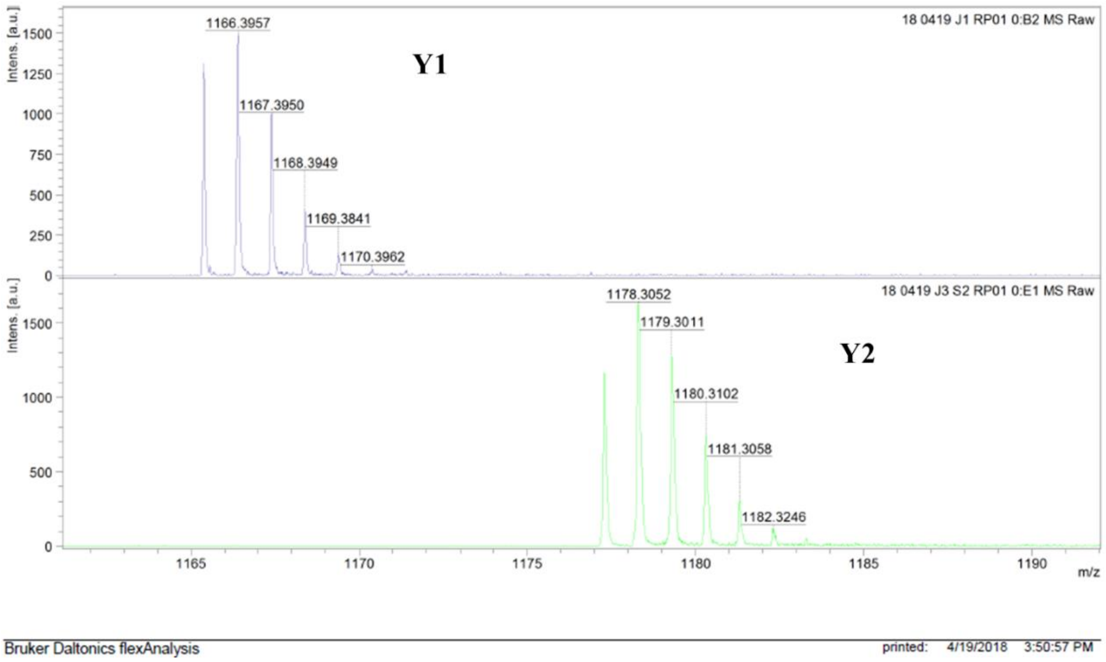

Supplementary Figure 8: The high resolution mass spectrum (MALDI-TOF) of Y1and Y2.

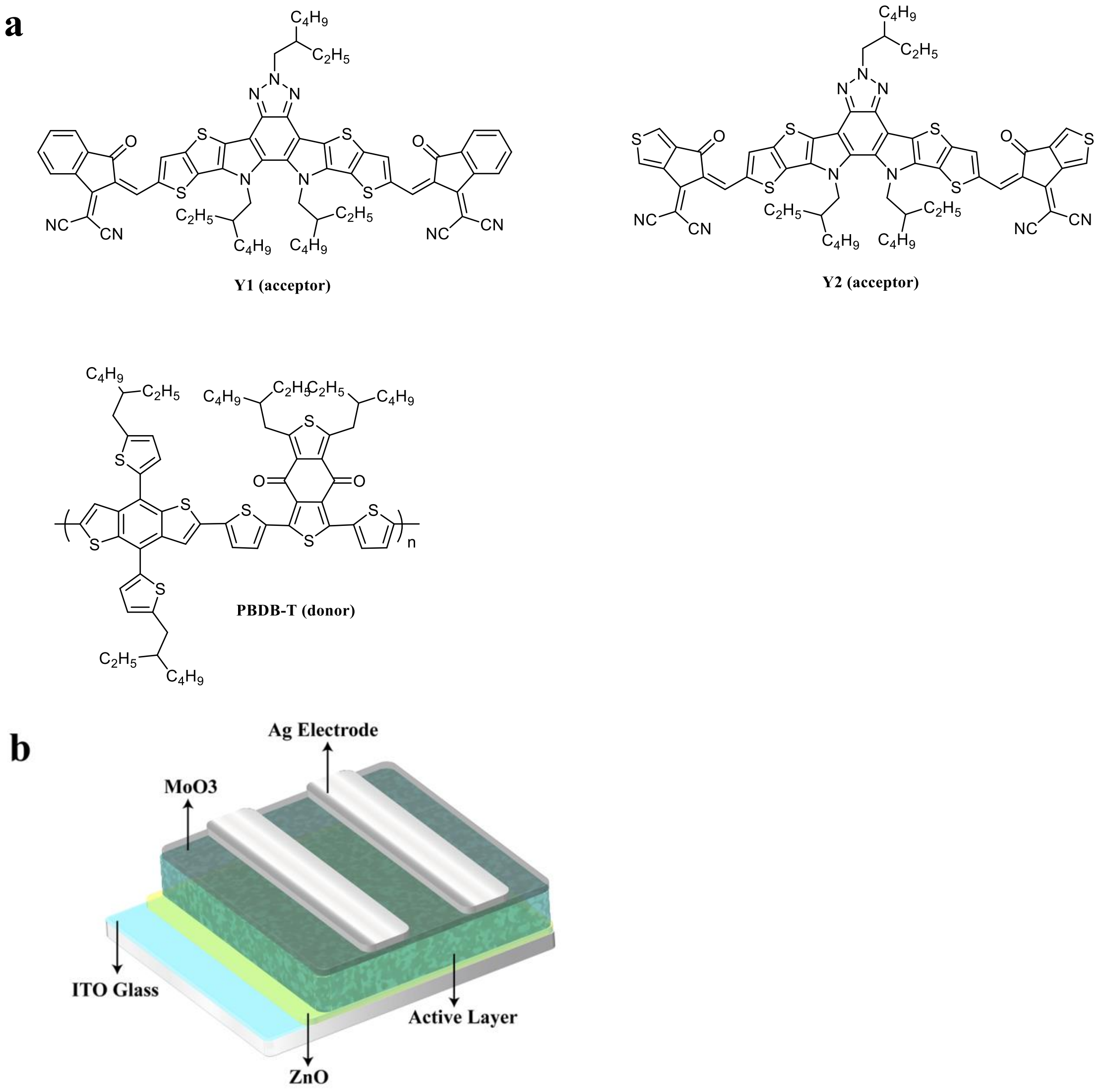

Supplementary Figure 9: a, Molecular structural formulas of the donor (PBDB-T) and the acceptors (Y1 and Y2) used. **b,** Device architecture of the solar cells.

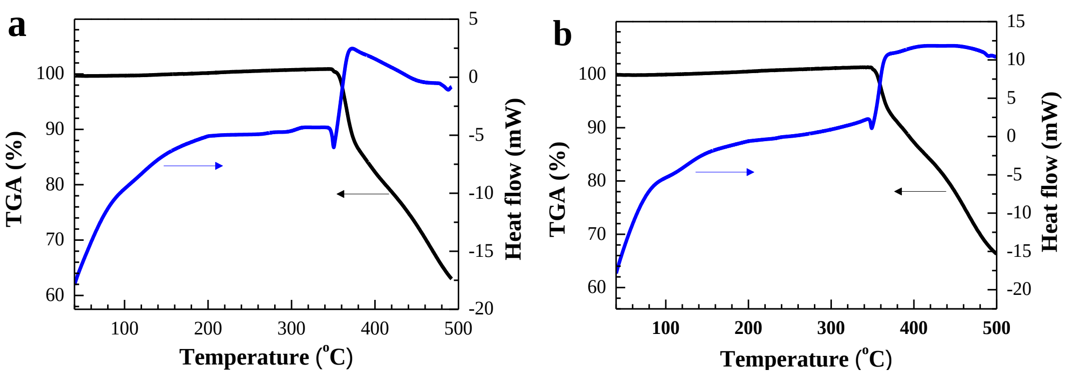

Supplementary Figure 10: Thermogravimetric analysis (black line) and heat flow (blue line) curves of **a**, Y1. **b**, Y2 with a heating rate of 20 K min^{-1}.

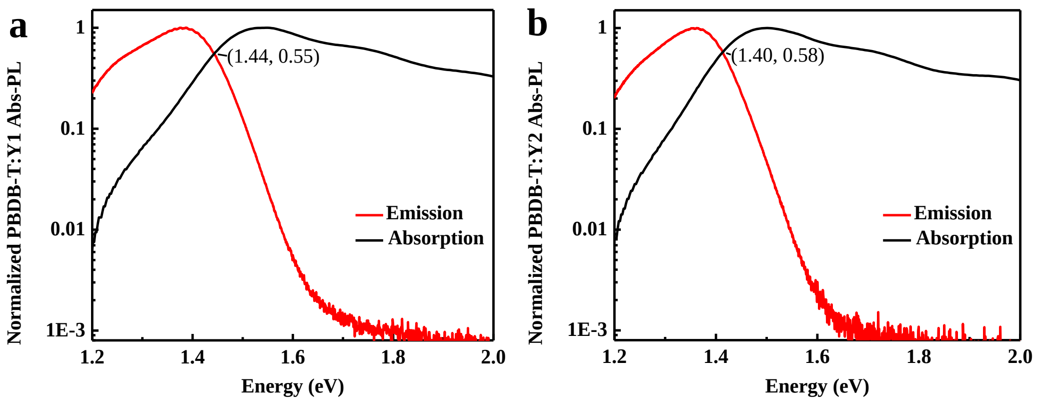

Supplementary Figure 11: Absorption and PL spectra of PBDB-T:Y1 or Y2 solar cells. The optical gap is determined at the intersection between two curves.

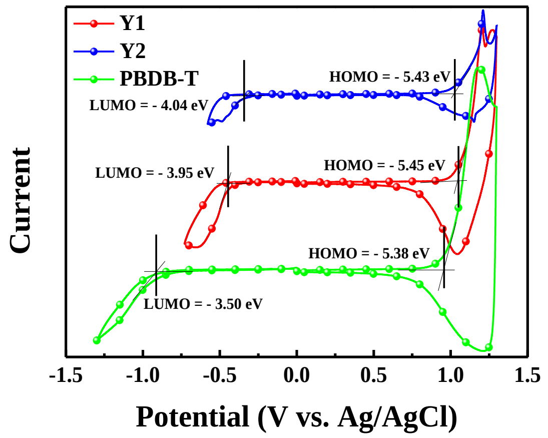

Supplementary Figure 12: Cyclic voltammograms of Y1, Y2 and PBDB-T films measured in 0.1 M Bu_4NPF_6 solution at the scan rate of 20 mV/s.

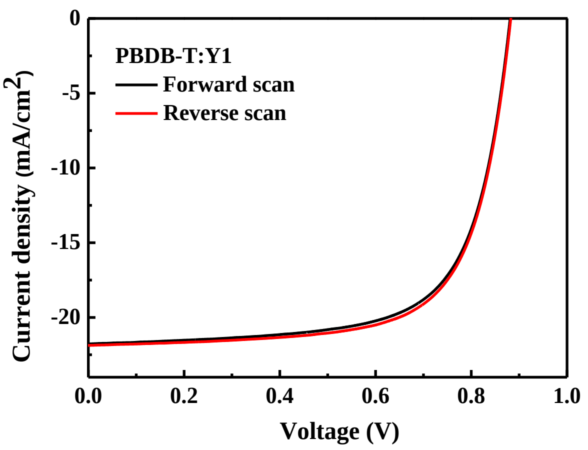

Supplementary Figure 13: J-V plots in both forward and backward direction of the cell based on PBDB-T:Y1.

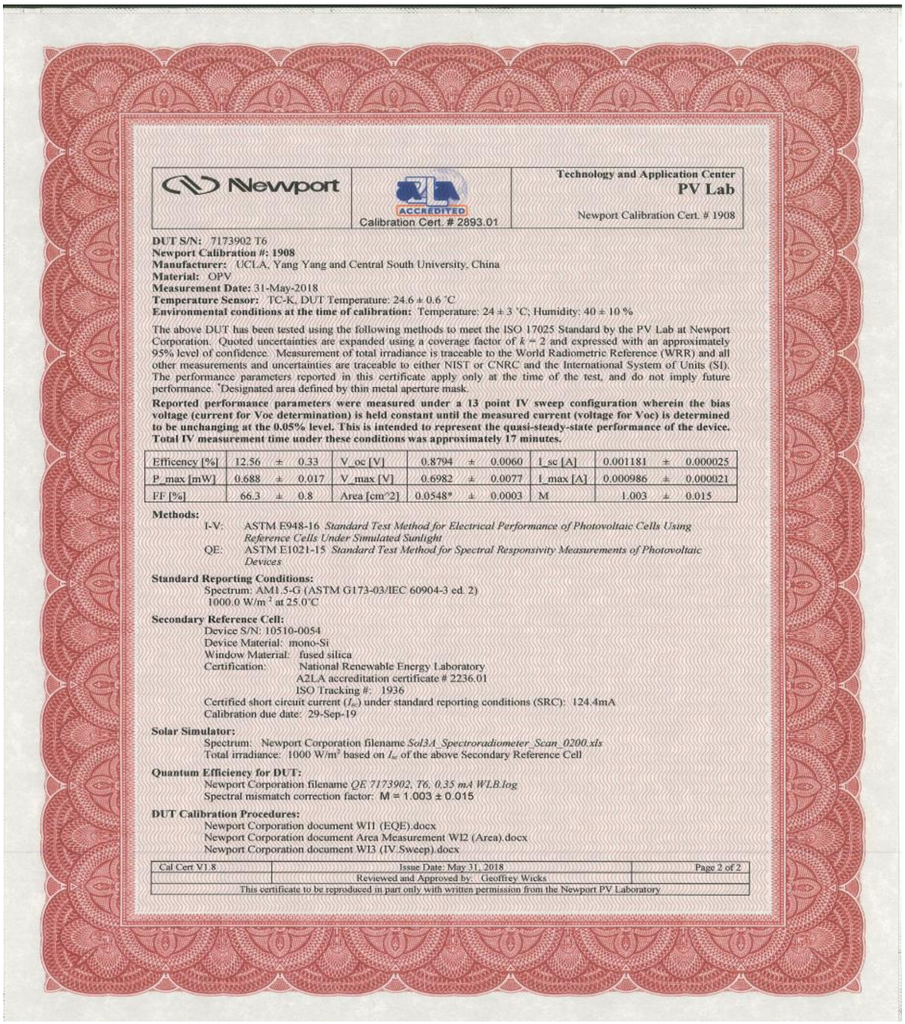

Technology and Application Center
PV Lab

Calibration Cert. # 2893.01

Newport Calibration Cert. # 1908

DUT S/N: 7173902 T6
Newport Calibration #: 1908
Manufacturer: UCLA, Yang Yang and Central South University, China
Material: OPV
Measurement Date: 31-May-2018
Temperature Sensor: TC-K, DUT Temperature: 24.6 ± 0.6 °C
Environmental conditions at the time of calibration: Temperature: 24 ± 3 °C; Humidity: 40 ± 10 %

The above DUT has been tested using the following methods to meet the ISO 17025 Standard by the PV Lab at Newport Corporation. Quoted uncertainties are expanded using a coverage factor of $k = 2$ and expressed with an approximately 95% level of confidence. Measurement of total irradiance is traceable to the World Radiometric Reference (WRR) and all other measurements and uncertainties are traceable to either NIST or CNRC and the International System of Units (SI). The performance parameters reported in this certificate apply only at the time of the test, and do not imply future performance. *Designated area defined by thin metal aperture mask.

Reported performance parameters were measured under a 13 point IV sweep configuration wherein the bias voltage (current for Voc determination) is held constant until the measured current (voltage for Voc) is determined to be unchanging at the 0.05% level. This is intended to represent the quasi-steady-state performance of the device. Total IV measurement time under these conditions was approximately 17 minutes.

Efficency [%]	12.56	±	0.33	V_oc [V]	0.8794	±	0.0060	I_sc [A]	0.001181	±	0.000025
P_max [mW]	0.688	±	0.017	V_max [V]	0.6982	±	0.0077	I_max [A]	0.000986	±	0.000021
FF [%]	66.3	±	0.8	Area [cm^2]	0.0548*	±	0.0003	M	1.003	±	0.015

Methods:
I-V: ASTM E948-16 *Standard Test Method for Electrical Performance of Photovoltaic Cells Using Reference Cells Under Simulated Sunlight*
QE: ASTM E1021-15 *Standard Test Method for Spectral Responsivity Measurements of Photovoltaic Devices*

Standard Reporting Conditions:
Spectrum: AM1.5-G (ASTM G173-03/IEC 60904-3 ed. 2)
1000.0 W/m^2 at 25.0°C

Secondary Reference Cell:
Device S/N: 10510-0054
Device Material: mono-Si
Window Material: fused silica
Certification: National Renewable Energy Laboratory
A2LA accreditation certificate # 2236.01
ISO Tracking #: 1936
Certified short circuit current (I_{sc}) under standard reporting conditions (SRC): 124.4mA
Calibration due date: 29-Sep-19

Solar Simulator:
Spectrum: Newport Corporation filename *Sol3A_Spectroradiometer_Scan_0200.xls*
Total irradiance: 1000 W/m^2 based on I_{sc} of the above Secondary Reference Cell

Quantum Efficiency for DUT:
Newport Corporation filename *QE 7173902, T6, 0.35 mA WLB.log*
Spectral mismatch correction factor: M = 1.003 ± 0.015

DUT Calibration Procedures:
Newport Corporation document WI1 (EQE).docx
Newport Corporation document Area Measurement WI2 (Area).docx
Newport Corporation document WI3 (IV.Sweep).docx

Cal Cert V1.8	Issue Date: May 31, 2018	Page 2 of 2
	Reviewed and Approved by: Geoffrey Wicks	
This certificate to be reproduced in part only with written permission from the Newport PV Laboratory		

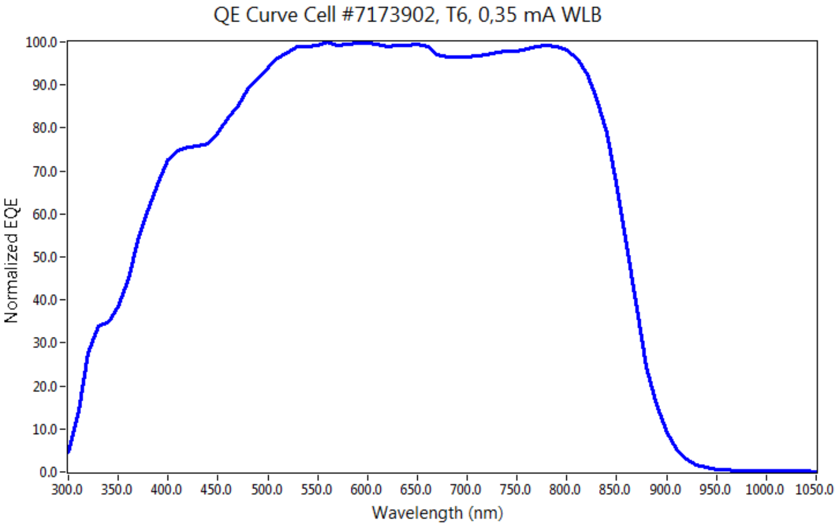

Supplementary Figure 14: Independent certification by Newport Corporation of PBDB-T:Y1 blend film solar cell confirming a high V_{oc} of 0.88 V and a stability power conversion efficiency of 12.56 %.

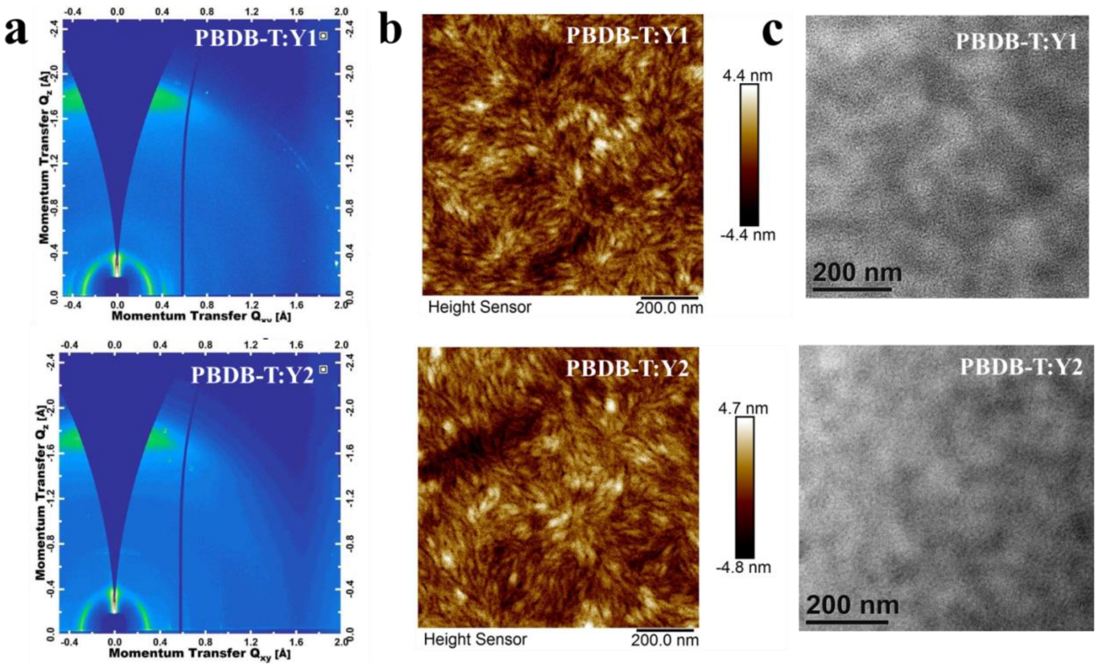

Supplementary Figure 15: Film morphology of PBDB-T:Y1 and PBDB-T:Y2 blend films. **a**, Two-dimensional GIWAXS images **b**, AFM images, and **c**, TEM images.

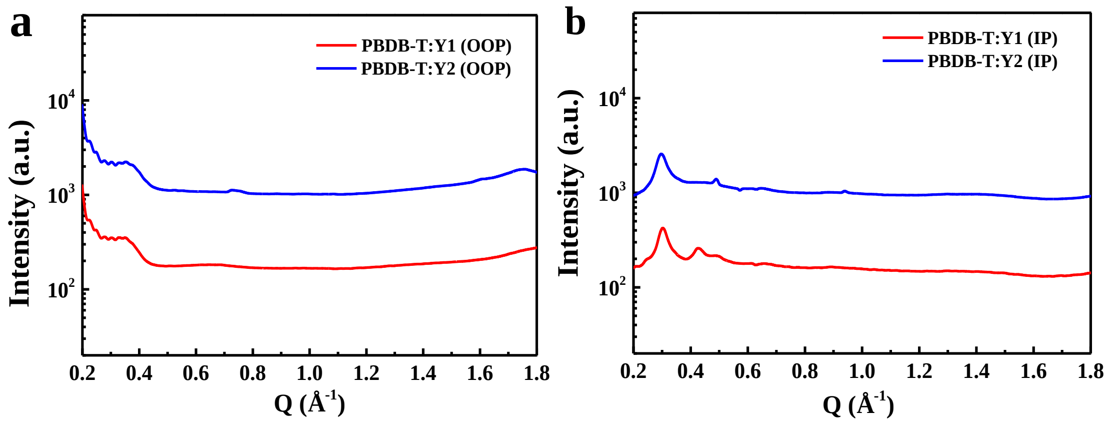

Supplementary Figure 16: a, Out-of-plane GIWAXS profiles for the PBDB-T:Y1 and PBDB-T:Y2 films. **b,** In-plane GIWAXS profiles for PBDB-T:Y1 and PBDB-T:Y2 films.

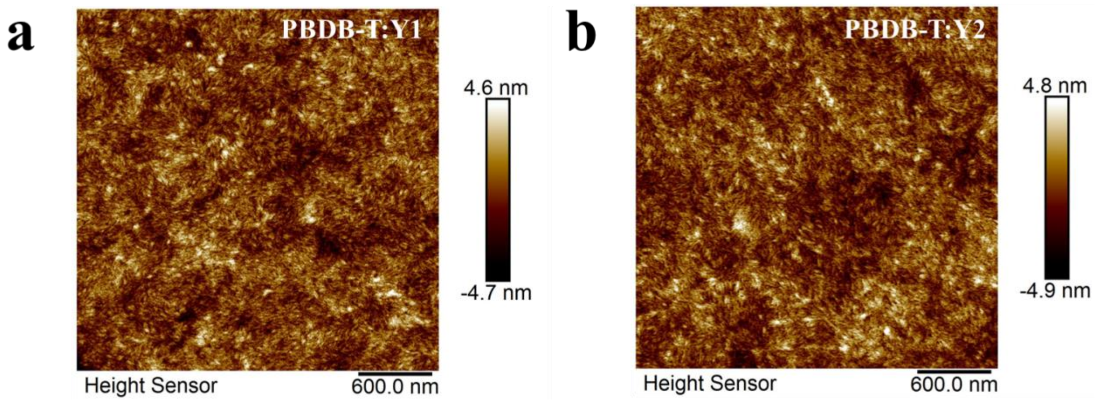

Supplementary Figure 17: AFM height images (0.6 μm × 0.6 μm) of the active layers: **a**, PBDB-T:Y1 film, and **b**, PBDB-T:Y2 film.

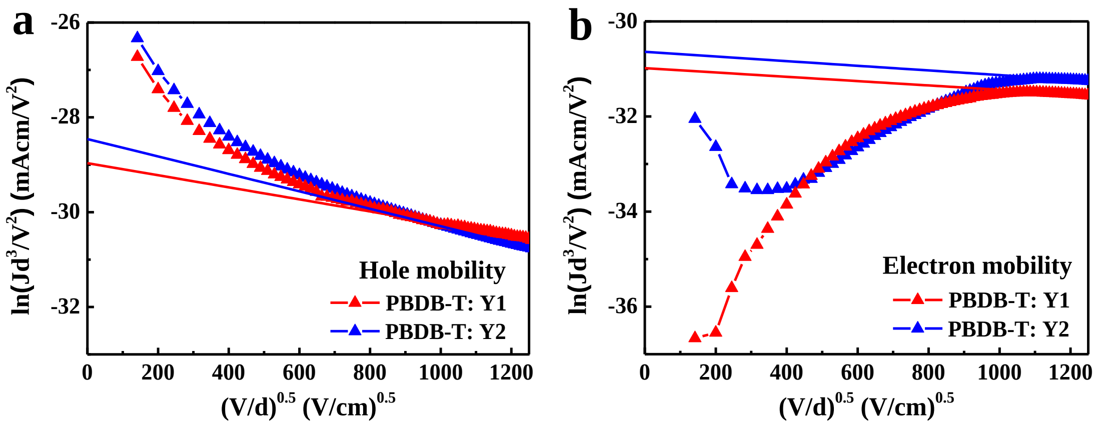

Supplementary Figure 18: a, Hole-only and **b,** electron-only devices based on PBDB-T:Y1 and PBDB-T:Y2 films.

Supplementary Tables

Supplementary Table 1. Optical and electrochemical properties of Y1 and Y2

	Absorption spectra				Cyclic voltammetry		
	Sol[a]	Film[b]			*p*-doping	*n*-doping	
	λ_{max} (nm)	λ_{max} (nm)	λ_{onset} (nm)	E_g^{opt} [c] (eV)	E_{on}^{ox} /HOMO[d] (V)/(eV)	E_{on}^{red} /LUMO[d] (V)/(eV)	E_g^{EC} (eV)
Y1	738	802	905	1.44	1.05 /-5.45	-0.45/-3.95	1.50
Y2	758	827	925	1.40	1.03/-5.43	-0.36/-4.04	1.39

a. Measured in chloroform solution.

b. Cast from chloroform solution.

c. The optical gaps from the intersection between normalized emission and absorption spectra.

d. HOMO= -e (E_{on}^{ox} +4.4) (eV); LUMO= -e (E_{on}^{red} +4.4) (eV) using (eV) using Ag/AgCl as the reference electrode.

Supplementary Table 2. Photovoltaic performances of different donor/acceptor ratios of PBDB-T:Y1 and PBDB-T:Y2 based PSCs devices with 16 mg mL^{-1} under 0.8% CN.

Devices	D/A ratio (*w/w*)	V_{oc} (V)	J_{sc} (mA cm^{-2})	FF (%)	PCE (%)
PBDB-T:Y1	1.2:1	0.90	19.62	67.6	12.1
	1:1	0.87	21.69	70.5	13.3
	1:1.2	0.86	19.38	68.6	11.5
	1:1.5	0.81	19.97	64.4	10.1
PBDB-T:Y2	1.2:1	0.85	18.92	66.5	10.7
	1:1	0.81	22.89	71.1	13.2
	1:1.2	0.79	21.78	69.7	12.0
	1:1.5	0.76	20.46	65.5	10.2

Supplementary Table 3. Summary of key parameters for the OPVs in recent papers[4-7].

**Binary devices**[a]	E_g (eV)	Voc (V)	PCE ($\%$)	ΔE (eV)	ΔE_1 $E_{gap} - qV_{OC}^{SQ}$ (eV)	ΔE_2 $q\Delta V_{OC}^{rad,\,below\ gap}$ (eV)	ΔE_3 $q\Delta V_{OC}^{non-rad}$ (eV)
PTB7-Th:SiOTIC-4F	1.25	0.65	7.0	0.60	0.26	0.05	0.29
PBQ-QF:IEICO-4F	1.36	0.74	10.5	0.62	0.27	0.06	0.29
PTB7-Th:COi8DFIC	1.36	0.70	10.5	0.66	0.27	0.04	0.35
PBDB-T:Y2	1.40	0.82	13.40	0.57	0.27	0.04	0.26
PBDB-T:Y1	1.44	0.87	13.42	0.57	0.27	0.05	0.25
PTB7-Th:IEICO	1.45	0.9	7.2	0.55	0.27	0.05	0.23
BTR:NITI	1.49	0.95	6.82	0.54	0.22	0.06	0.26
PNOz4T:$PC_{71}BM$	1.58	0.97	5.6	0.61	0.27	0.08	0.26
PBDB-T-2Cl:ITIC	1.61	1.04	9.2	0.57	0.28	0.03	0.25
PDCBT-2F:IT-M	1.67	1.13	6.4	0.54	0.28	0.05	0.21
PTB7-Th:$PC_{71}BM$	1.67	0.80	8.2	0.87	0.28	0.16	0.43
PvBDTTAZ:o-IDTBR	1.70	1.08	11.4	0.62	0.28	0.09	0.25
BTR:$PC_{71}BM$	1.82	0.90	9.03	0.92	0.29	0.23	0.40

Supplementary Table 4. The photoluminescence quantum yield of solid films based on Y1 and Y2 or blended with PBDB-T or polystyrene (PS).

	**PLQY of Y1 based film**	**PLQY of Y2 based film**
PBDB-T blend	0.1%	0.3%
PS blend	5.8%	3.4%
Pristine	5.2%	2.8%

Supplementary Methods

Spectrometry and spectroscopy: ^{1}H NMR and ^{13}C NMR spectra were recorded on a *Bruker* DPX 400 at 293 K. Chemical shifts (δ in ppm) were calibrated relative to solvent's residual proton and carbon chemical shift: $CHCl_3$ (δ = 7.26 ppm for ^{1}H NMR and δ = 77.0 ppm for ^{13}C NMR). Multiplicities of NMR signals are described as s (singlet) or m (multiplet). High resolution mass spectra were recorded on a Bruker Daltonics Micro Tof (ESI); peaks are given in m/z.

Chromatography: Thin layer chromatography (TLC) was performed using Merck silica gel 60 F-254 plates, detection of compounds with UV light (λ = 254 nm).

Materials: Unless otherwise noted, all chemicals were purchased from Sigma-Aldrich or Acros Inc. and used without further purification. PBDB-T was purchased from Solarmer Energy Inc. Toluene was dried over P_2O_5 and freshly distilled prior to use. All other reagents and solvents were purchased commercially as analytically pure and used without further purification. 4,7-Dibromo-2-(2-ethylhexyl)-5,6-dinitro- 2*H*-benzo[d]- [1,2,3]-triazole[1], tributyl(thieno[3,2-b]thiophen-2-yl)stannane[2] and 2-(6-oxo-5,6-dihydro-4*H*-cyclopenta[*c*]thiophen-4-ylidene)malononitrile[3] were synthesized according to previously reported methods.

Synthetic procedures and characterization

2-(2-ethylhexyl)-5,6-dinitro-4,7-bis(thieno[3,2-b]thiophen-2-yl)-2*H*-benzo[*d*][1,2,3]- trazole (3). Solution of 4,7-dibromo-2-(2-ethylhexyl)-5,6-dinitro-2*H*-benzo[d]- [1,2,3]-triazole (2.72 g, 5.7 mmol) and tributyl(thieno-[3,2-b]thiophen-2-yl)stannane (8.3 g, 19.8 mmol) was degassed in dry THF before, after which $Pd(PPh_3)_2Cl_2$ (0.24 g, 0.34 mmol) was added. After the mixture was refluxed under argon (Ar) for 12 hours, it was allowed to cool to room temperature and then concentrated under reduced pressure. The crude product was chromatographically purified on silica gel column with dichloromethane /hexane (1:4, *v/v*) as the eluent to afford compound **3** as an orange-red solid (2.8 g, 82 %).

^{1}H NMR (400 MHz, $CDCl_3$) δ 7.74 (s, 2H), 7.54 (d, *J* = 5.3 Hz, 2H), 7.33 (d, *J* = 5.9 Hz, 2H), 4.75 (d, *J* = 6.9 Hz, 2H), 2.27 (dd, *J* = 18.3, 12.2 Hz, 1H), 1.80–1.56 (m, 1H), 1.43–1.24 (m, 8H), 1.14– 0.72 (m, 6H).

6-(2-ethylhexyl)-12,13-dihydro-6*H*-thieno[2'',3'':4',5']thieno-[2',3':4,5]pyrrolo[3,2-*g*]- thieno[2',3':4,5]thieno[3,2-*b*][1,2,3]triazolo[4,5-*e*]indole (4). Compound **3** (1.80 g, 3 mmol) and triethyl phosphate (2.49 g, 15 mmol) were dissolved in *o*-dichlorobenzene (*o*-DCB, 30 mL). After refluxing under Ar overnight, the reaction was cooled to room temperature. The solvent was evaporated, leaving the precipitate (compound **4**), which was used without further purification.

6-(2-ethylhexyl)-12,13-dihydro-6*H*-thieno[2'',3'':4',5']thieno-[2',3':4,5]-pyrrolo[3,2-*g*]thieno[2',3':4,5]thieno[3,2-*b*][1,2,3]triazolo[4,5-*e*]indole (5). To a solution of compound **4**, potassium iodide (1.99 g, 1.2 mmol) and potassium carbonate (4.15 g, 30 mmol) in 30 mL N,N-dimethylmethanamide (DMF), under Ar atmosphere 1-bromo-2-ethylhexane (5.21g, 27 mmol) was added dropwise *via* a syringe. The mixture was heated to 90 °C overnight in the dark. The reaction solvent was removed under vacuum and extracted with ethyl acetate and water. The combined organic phases were dried over magnesium sulfate. After filtration, the solvents were evaporated under vacuum and the crude product (compound **5**) was purified on a silica gel using dichloromethane/hexane (1:5, *v/v*) as the eluent to give an orange-red solid (1.48 g, 65 %).

^{1}H NMR (400 MHz, $CDCl_3$) δ 7.41 (d, *J* = 5.2 Hz, 2H), 7.37 (d, *J* = 5.2 Hz, 2H), 4.73 (d, *J* = 7.1 Hz, 2H), 4.60 (d, *J* = 7.8 Hz, 4H), 2.40-2.33 (m, 1H), 1.98-1.89 (m, 2H), 1.43-1.26 (m, 8H), 1.01-0.86 (m, 16H), 0.61 (t, 6H), 0.53 (t, 6H).

6,12,13-tris(2-ethylhexyl)-12,13-dihydro-6H-thieno[2'',3'':4',5']-thieno[2',3':4,5]pyrrolo[3,2-g]thieno[2',3':4,5]thieno[3,2-b][1,2,3]triazolo[4,5-e]-indole-2,10-dicarbaldehyde (6). To a solution of compound **5** (0.99 g, 1.3 mmol) in dry DMF (20 mL) at 0 ℃, phosphorus oxychloride (1.8 ml, 19.5 mmol) was slowly added dropwise under Ar atmosphere. After stirring at the same temperature for additional 2 h, the solution was heated to 90 °C and stirred overnight. The reaction mixture was allowed to cool to room temperature and the mixture was poured into ice water, neutralized with Na_2CO_3 (aq), and then extracted with dichloromethane. After,

the collected organic layer was washed with water and brine. After removal of solvent, the crude product was purified using silica gel using ethyl acetate/hexane (1:5, *v/v*) as eluent, yielding a red solid of compound **6** (0.95 g, 89%).

^{1}H NMR (400 MHz, $CDCl_3$) δ 10.00 (s, 2H), 8.08 (s, 2H), 4.74 (d, *J* = 7.1 Hz, 2H), 4.63 (d, *J* = 7.7 Hz, 4H), 2.40-2.32 (m, 1H), 1.96-1.85 (m, 2H), 1.47-1.27 (m, 8H), 0.94 (dt, *J* = 14.2, 7.3 Hz, 22H), 0.64 (dd, *J* = 15.8, 11.0 Hz, 6H), 0.53 (dd, *J* = 15.3, 10.6 Hz, 6H).

2,2'-((2Z,2'Z)-((6,12,13-tris(2-ethylhexyl)-12,13-dihydro-6*H*-thieno-[2'',3'':4',5']thieno[2',3':4,5]pyrrolo[3,2-*g*]thieno[2',3':4,5]thieno[3,2-*b*][1,2,3]triazolo-[4,5-*e*]indole-2,10-diyl)bis(methanylylidene))bis(3-oxo-2,3-dihydro-1*H*-indene-2,1-diylidene))dimalononitrile (Y1). Under the protection of argon, compound **6** (0.19g, 0.23 mmol) and INIC (0.22g, 2.3 mmol) were dissolved in dry chloroform (25 ml), to which 1 mL pyridine was added after. After stirring at 70 ℃ overnight, the mixture was extracted with ethyl acetate and purified with column chromatography on silica gel using dichloromethane/hexane (3/1, *v*/*v*) as the eluent to give a blue-green solid (0.22 g, 83 % yield).

^{1}H NMR (400 MHz, $CDCl_3$) δ 9.02 (s, 2H), 8.71 (dd, *J* = 6.0, 2.2 Hz, 2H), 8.21 (s, 2H), 7.97 (dd, *J* = 5.7, 2.7 Hz, 2H), 7.81-7.72 (m, 4H), 4.74 (dd, *J* = 11.6, 7.5 Hz, 6H), 2.39-2.30 (m, 1H), 2.07-1.97 (m, 2H), 1.49-1.30 (m, 8H), 1.13-0.81 (m, 22H), 0.71 (t, *J* = 7.3 Hz, 6H), 0.60 (t, *J* = 7.1 Hz, 6H).

^{13}C NMR (101 MHz, $CDCl_3$) δ 188.49, 160.31, 143.03, 140.16, 138.12, 138.05, 137.87, 137.79, 136.89, 135.87, 135.13, 134.37, 133.18, 130.21, 125.30, 123.70,

121.91, 114.82, 111.93, 77.35, 77.03, 76.71, 68.91, 55.38, 40.28, 30.44, 29.63, 28.36, 27.63, 22.95, 22.73, 14.04, 13.67, 10.56, 10.24.

HR-MS (MALDI-TOF) *m/z* calcd. for ($C_{68}H_{63}N_9O_2S_4$): 1166.55. Found: 1166.3957.

2,2'-((5Z,5'Z)-((6,12,13-tris(2-ethylhexyl)-12,13-dihydro-6*H*-thieno-[2'',3'':4',5']thieno[2',3':4,5]pyrrolo[3,2-*g*]thieno[2',3':4,5]thieno[3,2-*b*][1,2,3]triazolo[4,5-*e*]indole-2,10-diyl)bis(methanylylidene))bis(6-oxo-5,6-dihydro-4*H*-cyclopenta[*c*]thiophene-5,4-diylidene))dimalononitrile (Y2). Y2 was obtained using a similar procedure used for the synthesis of Y1 starting from compound **6** (0.13 g, 0.16 mmol) and INTC (0.20 g, 1.0 mmol). Finally, the red solid was obtained (0.16 g, yield: 85 %).

^{1}H NMR (400 MHz, $CDCl_3$) δ 8.95 (s, 2H), 8.40 (d, *J* = 2.2 Hz, 2H), 8.20 (s, 2H), 7.98 (d, *J* = 2.2 Hz, 2H), 4.73 (dd, *J* = 7.2, 2.3 Hz, 6H), 2.35 (t, *J* = 7.2 Hz, 1H), 2.04-1.96 (m, 2H), 1.48-1.32 (m, 8H), 1.08-0.81 (m, 22H), 0.72 (dd, *J* = 12.6, 7.3 Hz, 6H), 0.58 (dd, *J* = 13.3, 6.1 Hz, 6H).

^{13}C NMR (101 MHz, $CDCl_3$) δ 181.60, 156.08, 142.67, 142.27, 139.40, 139.19, 139.16, 137.90, 135.86, 133.27, 130.43, 128.02, 127.49, 125.36, 115.19, 114.97, 114.51, 112.08, 77.35, 77.03, 76.71, 67.18, 55.41, 40.25, 30.44, 29.62, 27.58, 23.31, 22.95, 22.70, 14.03, 13.66, 10.55, 10.31.

HR-MS (MALDI-TOF) *m/z* calcd. for ($C_{68}H_{59}N_9O_2S_6$): 1178.60. Found: 1178.3052.

Supplementary Referneces

1. Feng, L. et al. Thieno[3,2-b]pyrrolo-Fused Pentacyclic Benzotriazole-Based Acceptor for Efficient Organic Photovoltaics. *Acs Appl Mater Interfaces* **9**, 31985-31992 (2017).
2. Kawabata, K., Takeguchi, M. & Goto, H. Optical Activity of Heteroaromatic Conjugated Polymer Films Prepared by Asymmetric Electrochemical Polymerization in Cholesteric Liquid Crystals: Structural Function for Chiral Induction. *Macromolecules* **46**, 2078-2091 (2013).
3. Zhang, Z. et al. Achieving over 10% efficiency in a new acceptor ITTC and its blends with hexafluoroquinoxaline based polymers. *J. Mater. Chem. A* **5**, 11286-11293 (2017).
4. Lee, J. et al. Design of Nonfullerene Acceptors with Near-Infrared Light Absorption Capabilities. *Adv Energy Mater* **8**, 1801209 (2018).
5. Liu, X. et al. Efficient Organic Solar Cells with Extremely High Open-Circuit Voltages and Low Voltage Losses by Suppressing Nonradiative Recombination Losses. *Adv Energy Mater* **8**, 1801699 (2018).
6. Nikolis, V.C. et al. Reducing Voltage Losses in Cascade Organic Solar Cells while Maintaining High External Quantum Efficiencies. *Adv Energy Mater* **7**, 1700855 (2017).
7. Zhou, Z. et al. High-efficiency small-molecule ternary solar cells with a hierarchical morphology enabled by synergizing fullerene and non-fullerene acceptors. *Nat. Energy* 3, 952-959 (2018)
